# Supplementary material for: Reaction Mechanism of CO2 with Choline-Amino Acid Ionic Liquids: A Computational Study
Source: Entropy (Basel). 2022 Oct 31;24(11):1572. doi: 10.3390/e24111572 (PMC9689648; doi:10.3390/e24111572)
Supplement: Supplementary file 1 [file entropy-24-01572-s001.zip › entropy-1954221-supplementary.pdf]

Article

Supplementary Materials: Reaction Mechanism of CO_2 with Choline-Amino Acid Ionic Liquids: A Computational Study

Fabio Ramondo [1,*] and Simone Di Muzio [2,3]

[1] Department of Chemistry, University of Rome 'La Sapienza', P.le Aldo Moro 5, I-00185 Rome, Italy
[2] Istituto dei Sistemi Complessi-Consiglio Nazionale delle Ricerche-ISC-CNR U.O.S. Sapienza, P.le A. Moro 5, 00185 Rome, Italy
[3] Department of Physical and Chemical Sciences, University of L'Aquila, Via Vetoio, I-67100 L' Aquila, Italy;
* Correspondence: fabio.ramondo@uniroma1.it

Keywords: CO2 capture; Ionic Liquids; DFT; Reaction mechanisms

Supplementary Materials

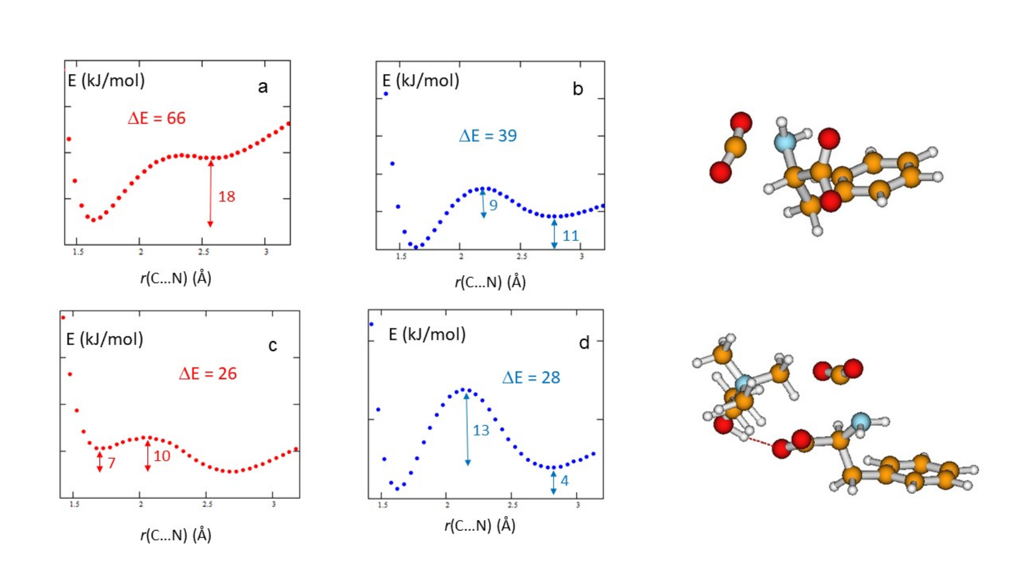

Figure S1. M062X/6-311++G** PES calculated at various C...N distances of [Phe] in vacuum (a) and PCM (b) and [Phe][Ch] in vacuum (c) and PCM (d) and M062X/6-311++G**(PCM) structure of the saddle points.
ΔE is calculated as $E_{[Phe-CO2]^-} - (E_{[Phe]^-} + E_{CO2})$
.

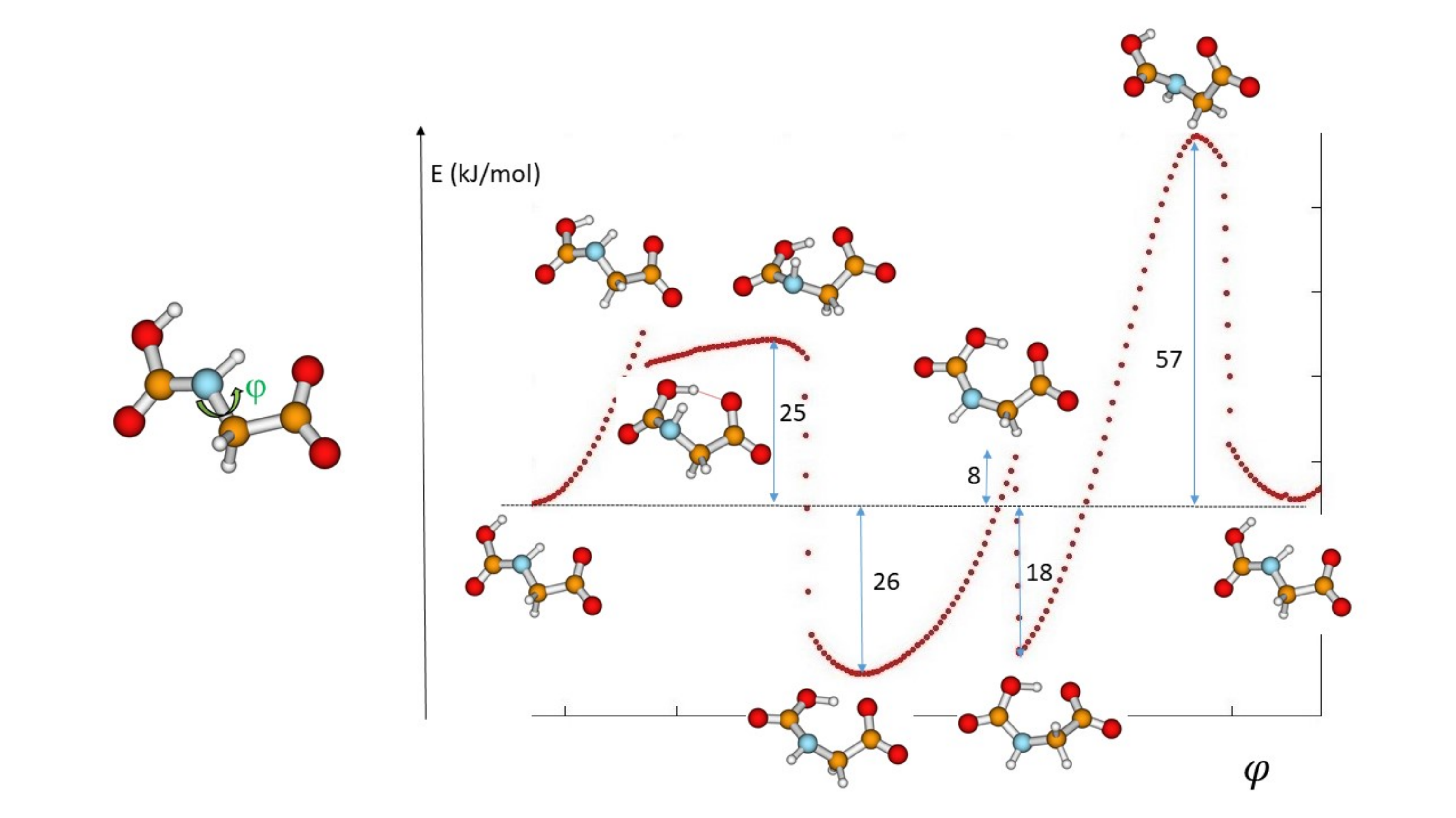

Figure S2. M062X/6-311++G** energy profile in function of the dihedral angle (φ) that describes the torsion about the C-N bond of the [Gly] anion calculated by PCM model. The stationary points localized in the PES scansion are reproduced in Figure.
